# Environment-Aware Rendering and Interaction in Web-Based Augmented Reality

**DOI:** 10.3390/jimaging9030063

**Published:** 2023-03-08

**Authors:** José Ferrão, Paulo Dias, Beatriz Sousa Santos, Miguel Oliveira

**Affiliations:** 1 Department of Electronics, Telecommunications, and Informatics (DETI), University of Aveiro, 3810-193 Aveiro, Portugal; 2 Intelligent System Associate Laboratory (LASI), Institute of Electronics and Informatics Engineering of Aveiro (IEETA), University of Aveiro, 3810-193 Aveiro, Portugal; 3 Department of Mechanical Engineering (DEM), University of Aveiro, 3810-193 Aveiro, Portugal

**Keywords:** augmented reality, immersive web, occlusion mapping, depth sensing

## Abstract

This work presents a novel framework for web-based environment-aware rendering and interaction in augmented reality based on WebXR and three.js. It aims at accelerating the development of device-agnostic Augmented Reality (AR) applications. The solution allows for a realistic rendering of 3D elements, handles geometry occlusion, casts shadows of virtual objects onto real surfaces, and provides physics interaction with real-world objects. Unlike most existing state-of-the-art systems that are built to run on a specific hardware configuration, the proposed solution targets the web environment and is designed to work on a vast range of devices and configurations. Our solution can use monocular camera setups with depth data estimated by deep neural networks or, when available, use higher-quality depth sensors (e.g., LIDAR, structured light) that provide a more accurate perception of the environment. To ensure consistency in the rendering of the virtual scene a physically based rendering pipeline is used, in which physically correct attributes are associated with each 3D object, which, combined with lighting information captured by the device, enables the rendering of AR content matching the environment illumination. All these concepts are integrated and optimized into a pipeline capable of providing a fluid user experience even on middle-range devices. The solution is distributed as an open-source library that can be integrated into existing and new web-based AR projects. The proposed framework was evaluated and compared in terms of performance and visual features with two state-of-the-art alternatives.

## 1. Introduction

Augmented reality (AR) consists in augmenting the real world with virtual layers of information [1]. Since its introduction, AR has grown substantially and is now used in several well-known applications such as Facebook, Snapchat, Google Maps, and Pokémon Go. Common applications include AR-powered photography filters, visual map navigation assistance in vehicles, or even more complex tasks such as remote technical assistance [2] and medical surgery training [3].

While all AR systems need pose tracking to align virtual content with the environment, most systems still do not possess the capability of dynamically adjusting content to their surroundings, limiting the immersion of the AR application, an essential factor for the effectiveness of these systems as discussed by [4].

Environment awareness in AR systems is an important step not only from an interactive and immersive perspective but also to improve usability, for example by adjusting fonts or content colour to improve readability based on the environment, as studied by [5].

The computing power of mobile devices has improved significantly over the last decade and these devices have become the most common scenario for AR applications. Currently, mobile devices have powerful systems on a chip (SoC) that contain motion sensors capable of good position and orientation tracking, which, paired with modern visual camera systems composed of multiple image sensors, make smartphones a natural fit for AR experiences.

Although smartphones are the most common type of devices used for AR, advanced features and more precise tracking are available on dedicated wearable devices, such as Microsoft HoloLens 2 [6], Magic Leap, or the Real Wear HMT-1. These devices provide a hands-free experience essential for the adoption of AR in many scenarios such as industrial environments. Head-mounted displays (HMD) can also be equipped with hardware for eye tracking [7] and hand tracking [8], enabling natural interaction with the virtual world. HMD allow for a foveated rendering [9,10], a technique to improve computing efficiency by reducing rendering detail in the peripheral vision of the user.

To accelerate the development of AR applications, vendors have introduced dedicated APIs (e.g., Google ARCore, Apple ARKit) that simplify the integration of advanced hardware features that provide details about the environment in which the user is located (e.g., geometrical information, lighting conditions, device tracking, environment landmarks).

Web-powered AR offers the capability of a setupless experience, without the need to download and install applications while offering developers the possibility of developing cross-platform AR experiences. Using modern application programming interfaces (API) (e.g., WebGL, web workers, WebGPU), web-based AR experiences can achieve performance close to native applications, while being faster to develop due to the simplified nature of the web ecosystem.

**Figure 1:**
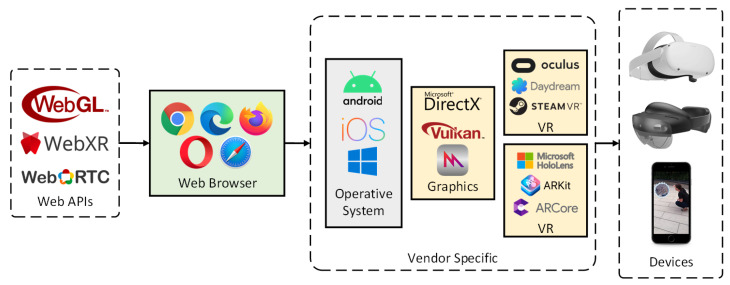
Web ecosystem for the development of cross-platform AR applications; web APIs get translated into vendor-specific APIs by the web browser.

WebXR [11] is a web-based API that works as middleware between the web layer and the dedicated system APIs available across multiple devices, providing a base for both AR and virtual reality (VR) development using web technology. It allows for the development of AR applications that are device agnostic and that can work on smartphones and dedicated extended reality (XR) devices. It allows developers to target multiple devices (as exemplified in Figure 1) and provide fallback code for users that access the application without an AR-capable device, important for example to provide access to users with disabilities [12].

With the rise in real-time collaborative AR experiences, the usage of web technologies has become more relevant as they allow for cross-platform communication and collaboration from a single application. Works such as the ones proposed by [13] or [14], where the authors present a solution for remote conference with support for AR and VR, explore the versatility of the web ecosystem.

In this document, a novel framework for environment-aware AR applications based on WebXR is presented. The novelty of this work stems from the integration of many state-of-the-art concepts in AR such as occlusion rendering, environment lighting, shadows, and physics interaction between virtual and real objects into a single performant pipeline.

**Figure 2:**
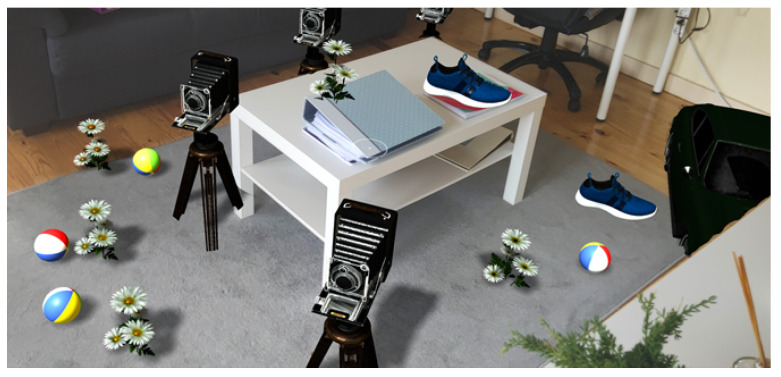
Environment-aware augmented reality rendering with geometry occlusion, shadow casting, lighting matching, and physics interaction between virtual and real world objects.

The solution uses physically based shading, combined with environment information that results in the rendering of 3D objects that are consistent with the real-word surroundings as demonstrated in Figure 2. The performed tests confirm that the proposed approach can achieve 30 fps on a middle-range device while running directly from the web browser.

This work was motivated by the lack of cross-device AR solutions that provide environment-aware rendering and physics for AR. Current solutions for cross-device AR (e.g., Vuforia, ARToolKit+, Playcanvas) are limited to basic AR functionalities such as tracking and scene alignment [15].

The rest of the paper is structured as follows: Section 2 introduces and discusses the related work regarding environment-aware AR systems; Section 3 presents the technical approach for web-based environment-aware AR; in Section 4, the performance of the framework implemented is analysed; in Section 5, the performance evaluation of the framework is presented and compared with other state-of-the-art solutions; and Section 6 concludes the work and proposes some ideas for future developments on the topic presented.

## 2. Related Work

### 2.1. System-Level API

Traditional AR applications require previous knowledge of the environment or the usage of discrete markers to align the virtual information with real-world images and provide adequate tracking between the virtual and real worlds.

The search for solutions capable of mapping and recognizing the environment for AR applications has been a topic of research for as long as the AR term has existed; an early solution for environment interaction in AR with depth estimation from a stereo camera was presented in [16].

In 2014, Google introduced the Tango SDK, used to build AR devices equipped with depth sensors and motion tracking, capable of obtaining a detailed 3D mapping of the environment using simultaneous location and mapping (SLAM) algorithms. This innovative platform kickstarted the growth in mobile environment-aware AR and opened new possibilities regarding environment tracking and geometry occlusion between real and virtual worlds [17,18].

In 2017, Apple introduced a concurrent to Google Tango, the ARKit, which used the already existing hardware of iPhone and iPad devices for environment tracking, depth sensing, and lighting information.

In 2018, an efficient algorithm for the densification of depth points obtained from monocular visual feature tracking integrated into a SLAM process using deep neural networks (DNN) capable of running in real time was proposed by [19]. It produced depth data consistent with the edges in the colour images used as reference for the densification, specifically targeting a usage in AR applications paving the way for massification of cost-effective AR solutions without the requirement of expensive depth systems by leveraging the AI capabilities already embedded into modern mobile SoC.

In the same year, Google introduced a new API for AR applications named ARCore. As its counterpart (Apple ARKit), ARCore could run on already available smartphones by using DNN to densify depth data obtained from a single camera alongside with phone position and orientation provided by the inertial measurement unit (IMU). It was also compatible with dedicated depth hardware such as time-of-flight (ToF) cameras that can provide more precise depth information. Alongside with device tracking and environment geometry information, ARCore also introduced the possibility of obtaining lighting information, image tracking, human face recognition, and mechanisms for real-time collaborative AR among devices by sharing environment features across devices [20].

Although ARCore and ARKit proposed similar functionality, there were differences in their technical approach that were investigated by [21,22], where the authors explored the performance and quality of these APIs for tracking in indoor and outdoor scenarios.

In 2020, a solution for environment-aware AR named DepthLab [23] was introduced. It leveraged depth and lighting information provided by ARCore to build environment-aware AR experiences with geometry occlusion, real environment relighting, and physics interaction.

Also in 2020, Apple introduced solid-state Light Detection and Ranging (LIDAR) technology in smartphones. LIDAR provides high-accuracy dense depth data without the need for additional processing from DNN, improving both performance and quality of the depth information. Two examples of applications using this solution are Snapchat, which provides accurate geometry occlusion and relighting of a real world image, and Polycam, which uses the geometry provided from the LIDAR to perform a 3D reconstruction using SLAM.

### 2.2. Web-Based AR

The web environment offers one of the most complete toolsets for Graphical User Interface (GUI) development with the possibility of developing complex 3D systems using WebGL.

AR.js was the first framework widely adopted for web AR targeting smartphone devices with marker-based tracking. This approach requires the usage of markers such as QRCode, Aruco, or pretrained images to track the environment using the device camera.

The usage of markers with data storage also provides context for the AR system (e.g., markers can be used to dynamically reference 3D models from a server). Without markers, AR systems must rely on object detection and classification techniques that can be computationally expensive for mobile devices.

With the introduction of the first versions of WebXR, markerless web-based AR solutions have started to appear in scientific works and commercial applications. However, while WebXR provides the required technology to implement AR experiences, the inherent limitation of the web environment regarding data bandwidth and the complexity of leveraging the high-performance functionality still impose a barrier for fully featured experiences leading to badly implemented solutions, as studied in [24].

In 2020, a solution for AR interaction with point cloud data using three.js and WebXR for environment tracking and interaction was proposed, providing a limited but performant example of a web application [25].

### 2.3. Realistic Rendering

The real-time realistic rendering of virtual images has been a research topic widely explored over the years from simulation applications, gaming systems, and even industrial applications. In the last few years, we observed an exponential increase in the processing capabilities of graphics hardware. Ray tracing is now a viable solution for hybrid rasterized systems in desktop computers and gaming consoles allowing the simulation of paths of light. Low-power mobile devices are now also capable of realistically rendering images using techniques that were previously not feasible such as physically based rendering (PBR) or the usage of advanced screen-space effects [26].

AR realistic rendering techniques have been constrained by the lack of environment information (e.g., lighting conditions, geometry) and limitations in performance. In 2003, image-based lighting (IBL) [27] was introduced. IBL is a technique for photorealistic rendering using environment maps for object shading and reflections in AR. Later in 2004, a solution [28] for shadow rendering in AR was proposed using shadow volumes, a prebuilt model of the environment geometry, and manually tweaked lighting. Both these solutions required previous knowledge of the environment and markers for environment tracking but provided credible results despite some limitations.

In 2019, DeepLight [29], a DNN-based solution capable of estimating scene illumination from a single photograph with a low dynamic range (LDR) and a limited field of view, was proposed. The technique used a DNN trained with pictures of the environment and high-dynamic-range (HDR) maps obtained for three different physical material samples. Based on a single picture, the DNN generated an HDR map. This map could be mapped to the objects’ surface using IBL rendering.

## 3. Proposed Solution

### 3.1. Framework Development

For the implementation of the solution, the three.js graphics library and the JavaScript programming language were selected. The three.js library was chosen based on its rendering capabilities with PBR and WebGL 2.0 support and on its popularity to increase the reach of the solution proposed to researchers and developers. Three.js has a much larger community of users when compared to the closest competitors Babylon.js and Play Canvas.

For GUI elements, the “dom-overlay” functionality, which allows the integration of document object model (DOM) elements in the AR scene, was used to implement an abstraction layer that projected and aligned these elements into the 3D scene using CSS transforms extracted from the camera projection model. These elements could also be used in 2D for on-screen user inputs and information.

For the development and tests, a Xiaomi Mi 10 Lite 5G (M2002J9G) smartphone (manufactured by Xiaomi Inc in China and sourced from Porto, Portugal) was selected as a representation of a middle-range device that supports ARCore depth functionality. The device was equipped with a Qualcomm 765G SOC and 6 GB of RAM using the operating system Android 10. The code was tested using Chrome 88.0.4324.68. Models used for testing were obtained from the Khronos Group glTF 2.0 sample repository.

### 3.2. Object Picking

In this section, the object-picking module of the solution used for interaction with the 3D virtual environment from a 2D screen points using the “hit-test” functionality of WebXR is presented. The hit-test system assumed that all interactions were performed over plane surfaces; it was capable of handling multiple planes simultaneously and provided the normal of the plane surface. The API received a ray described by its origin and direction vector and responded with the estimated distance to a real-world surface, if any. Points obtained from the hit-test were stored internally by the API as environment anchors used to realign the scene when the tracking was lost, ensuring the objects placed in the scene were always correctly realigned after tracking problems.

The object placement mechanism was used to introduce virtual 3D objects using a position calculated from the hit-test result. The solution allowed the user to select screen-space points for which the origin and direction of the ray were calculated based on the device position (obtained from tracking) and the camera projection characteristics.

Object picking could be combined with dedicated controllers using the Gamepad API and motion-sensing-enabled controllers (e.g., Wii Remote) to further improve immersion. Using the object placement module, we also implemented sample measurement tools to evaluate distances, angles, and areas using the GUI toolkit.

**Figure 3:**
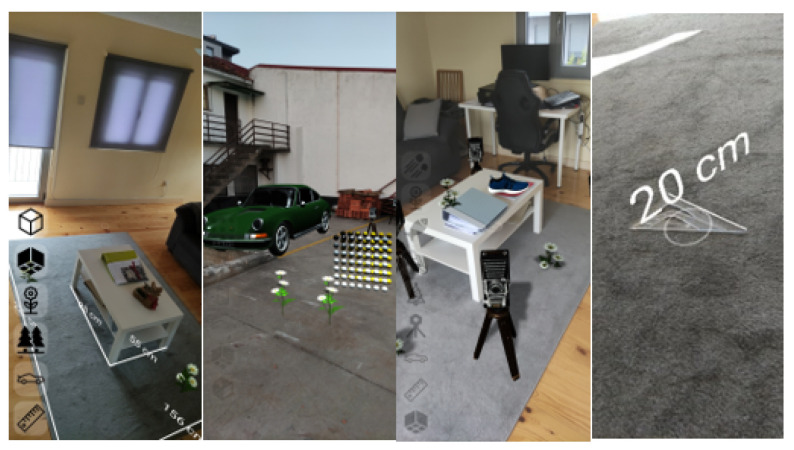
Object placement and measurement functionalities.

The accuracy of the tracking solution provided by the API was analysed in depth by [30], where the authors observed a standard deviation of up to 0.73 cm but also observed a high dependency on the environment’s visual features and lighting conditions. Although a consistent test environment was not used, it was possible to observe measurements of objects with a known size as exemplified in Figure 3 (right) by measuring a 20 cm ruler with an accuracy of 1 cm, with the device positioned 40 cm away from the object. The maximum observed depth captured by the system was 8 m.

### 3.3. Geometry Occlusion

**Figure 4:**
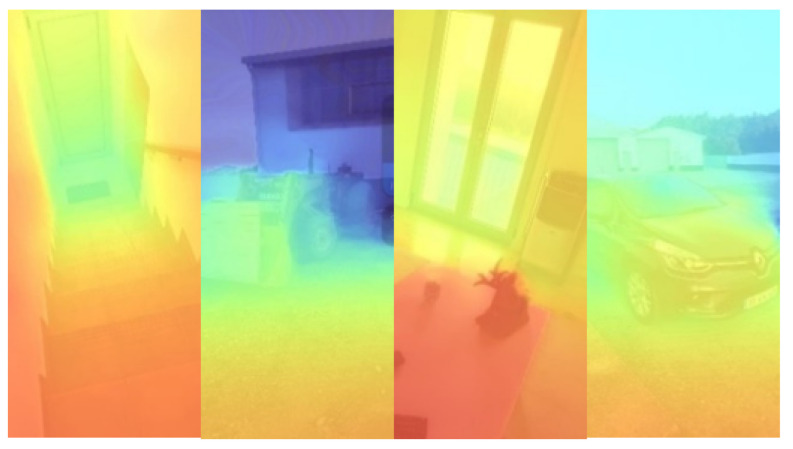
Depth data estimated from monocular views (closer surfaces in red and surfaces further away in blue).

In this section, a Graphics Processing Unit (GPU) based solution for object occlusion is presented. The quality of the depth data available depends on the system configuration and sensors existing on the device. The approach used allowed for high-precision depth data when available, by integrating the raw depth data into the rendering pipeline. The test device used a monocular depth estimation provided by the AR Core obtained using a DNN-based algorithm [23] with a resolution of 160×190 pixels and up to 8 m in distance exemplified in Figure 4.

**Figure 5:**
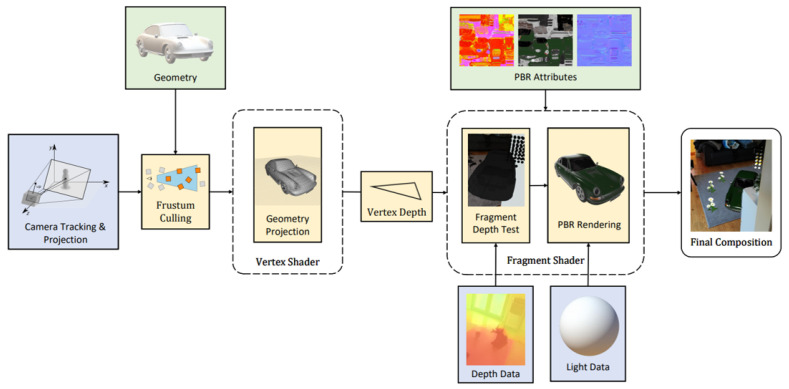
Rendering pipeline for geometry occlusion: the vertex shader calculates the projected vertex depth and passes it to the fragment shader where the depth of each fragment is evaluated against the depth data. Only visible fragments are rendered; all geometries are frustum culled to prevent any computation on geometries that are completely outside of the camera viewport.

The rendering pipeline for the geometry occlusion is presented in Figure 5. Tracking information was used to align the virtual scene with the real-world environment. Before starting the rasterization process, frustum culling [31] was used to remove any objects outside of the viewport. After rasterization, the normalized depth information was tested with each fragment, and portions of the scene that would appear over the real-world environment were discarded.

The depth data were provided as an array of 16-bit depth values, efficiently stored in a WebGL texture using the luminance alpha mode, which allowed for two 16-bit unsigned byte colour channels, exactly matching the size of the input data. The depth data provided were not normalized by default so the depth buffer coordinates may not correspond to screen-space coordinates. To convert from depth buffer coordinates, a normalization matrix was provided by the API that had to be applied to correct the screen coordinates.

**Figure 6:**
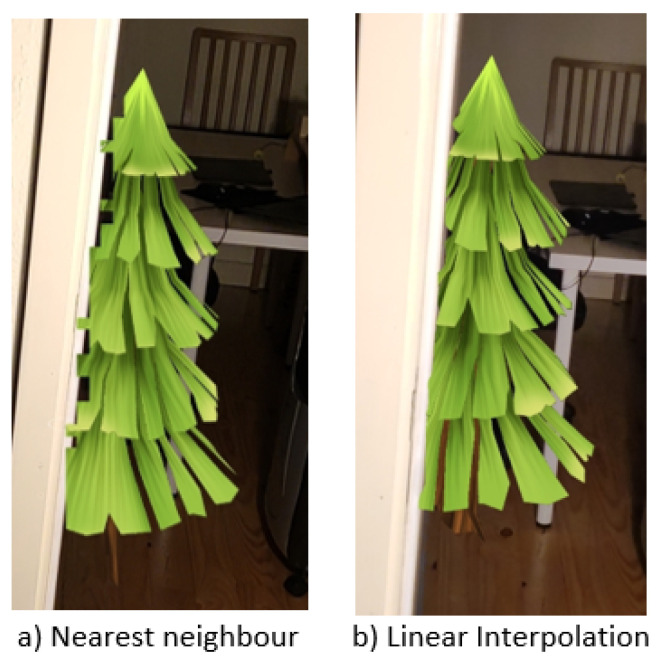
Comparison between nearest neighbour (**a**) and linear filtering (**b**) for depth data resampling.

The resolution of the depth data could be different from the screen resolution depending on the system. In the used test scenario, the depth resolution of 160×190 pixels was much smaller than the image resolution. A linear interpolation was used to filter and upscale the depth data to match the rendering resolution. Figure 6 presents the results obtained with the linear interpolation compared to a nearest-neighbour rescaling, which leads to a cleaner depth in the image on the right. Upscaling of the depth data was performed in the shader by improving the depth occlusion without any noticeable performance impact.

To reuse the existing materials from the three.js library, we analysed how the materials shaders were built from the material description. The shaders were built from a set of OpenGL Shading Language (GLSL) shader chunks, and each material had its own shader program, which, after being compiled, was managed by the internal renderer of the library.

The shader code generated could be accessed from the “onBeforeCompile” call-back executed before the GLSL code was compiled, where the code for the depth occlusion was injected into the existing shader code. In that phase, uniform variables could be registered, specifically, the depth texture shared between all shaders, the normalization matrix, and the screen size. These uniforms were mapped from their JavaScript attribute name to the shader code exactly and had to be managed manually to prevent memory leaks.

To blend the result between virtual environments and real-work images, a preprocessing step was applied to prevent the overdraw of fragments that would be latter discarded. To ensure no loss in precision, we compare the depth data encoded in data texture against the projected vertices z passed to the fragment shader. This approach avoided the truncation of the precision of the depth data to match the depth buffer accuracy.

The screen-space depth coordinates were obtained through the rectification matrix to reconstruct the 16-bit depth value from the two 8-bit components, obtaining the original depth value in millimetres. Any fragments that had a lower depth value, meaning that they were further away from the projection origin than the real-world surface, were discarded from the result before any additional processing was performed.

**Figure 7:**
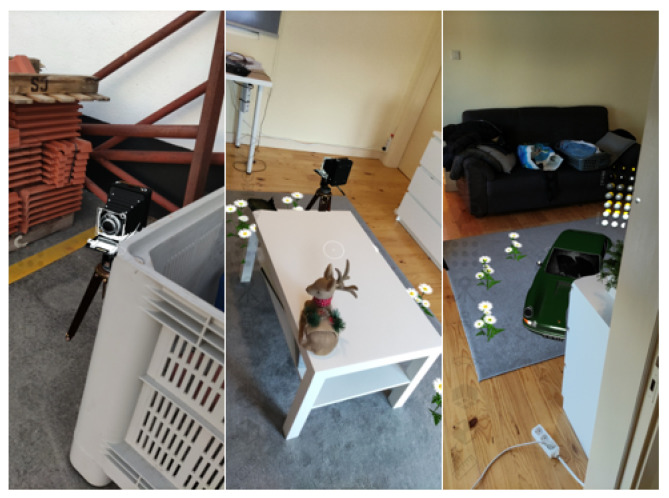
Depth occlusion between real-world and virtual objects.

Combining real and virtual objects using the method proposed provided visually accurate results, where it was possible to distinguish the separation between real and virtual objects across their borders as shown in Figure 7 for indoor and outdoor scenarios.

**Figure 8:**
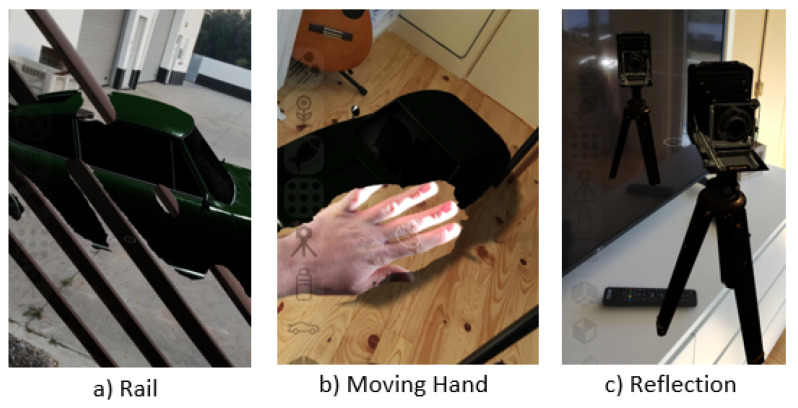
Inconsistency in depth data and poor occlusion results.

For some scenarios, it was possible to observe some inconsistencies due to errors in the depth data observed with virtual objects placed behind objects with holes, translucid objects, or with the presence of highlights and reflective surfaces and moving objects. Figure 8 shows three examples of these inconsistencies: (a) shows a depth failure in a perforated object; (b) the movement of the user hand is perceived as a solid object; and (c) the depth confusion is caused by the reflective TV surface, allowing the user to place objects inside the TV reflection.

### 3.4. Lighting

This section describes how the solution uses lighting information and PBR rendering to provide a consistent shading appearance with the environment. PBR rendering consists in using physical models to calculate lighting in 3D-rendered scenes, instead of using a visually obtained approximation to model light interaction with surfaces. The idea is that instead of tweaking materials to “look good” under specific lighting, a material can be created that will react correctly according to calibrated lighting scenarios. In practice, this gives a more accurate and realistic-looking result than the Lambert or Blinn–Phong [32] models.

Bidirectional reflectance distribution functions (BRDF) [33] define how light is reflected at any point of a surface based on its physical properties (e.g., metalness, roughness, emissivity), and are used to describe the PBR shading model.

The “lighting-estimation” module of WebXR provides light intensity, the direction of the main light source, and a cube-map texture of the environment surroundings. These data are obtained from a device’s light sensor and camera.

To access lighting data, a light probe was used to query the direction of the main light source as well as environment light information provided as a set of spherical harmonics (SH) [34] at each frame.

SH encode lighting conditions in spherical functions that are then projected into frequency space and represented by 9 coefficients. It is an alternative representation to a full cube map that uses less memory and is effective for scenarios where high-frequency details are not required. The API provides a set of SH coefficients for each RGB colour channel.

The three.js library has a PBR rendering implementation based on the model described in [35]. The SH information retrieved was mapped to the three.js light probe model, and light intensity and direction were represented as a directional light.

**Figure 9:**
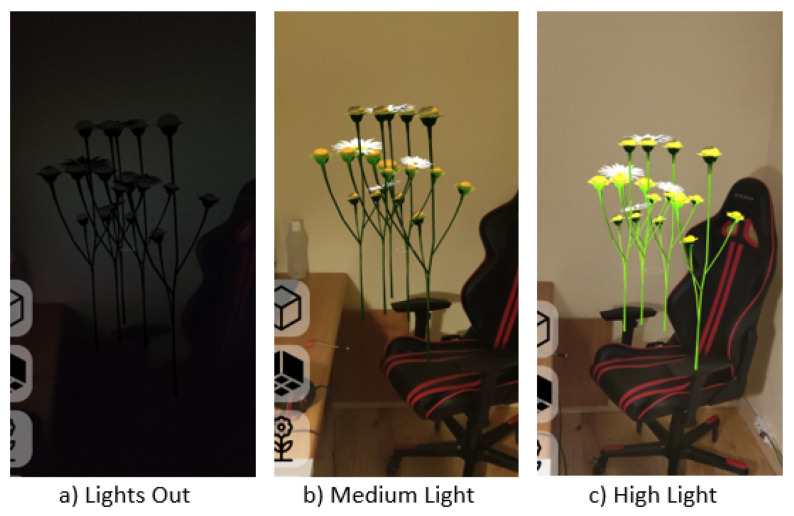
Virtual object reacting to different environment lighting conditions.

The 3D models used in the framework must contain all the required physical attributes of their surfaces encoded into textures. Figure 9 presents the results, showing flowers reacting to the varying lighting conditions in the room matching the direction, intensity, and tonality of the light.

### 3.5. Shadows

Using the lighting information, the framework provided a solution for casting AR shadow between 3D objects and the ground planes. The technique described in Section 3.2 was used to sample the environment at every frame to scan for planes visible in the screen space.

Planes found were used to render shadows using shadow maps [36] cast from the light sources to the planes detected in the environment. This technique allowed objects placed on different surfaces to cast shadows onto any planar surface (e.g., ground, tables, walls). To prevent shadows from being incorrectly projected over real-world objects, depth was considered to clip the shadow maps based on the surface distance to the camera.

The usage of shadow maps presents some limitations: it is not possible to have partial light occlusion from translucid objects, shadows are projected for a finite area, and the number of lights casting shadows must be limited, since computational cost and memory increase exponentially with the number of lights in the scene. Numeric precision limitation may also cause a shimmering effect commonly known as “shadow acne”, that was studied and addressed in [37].

**Figure 10:**
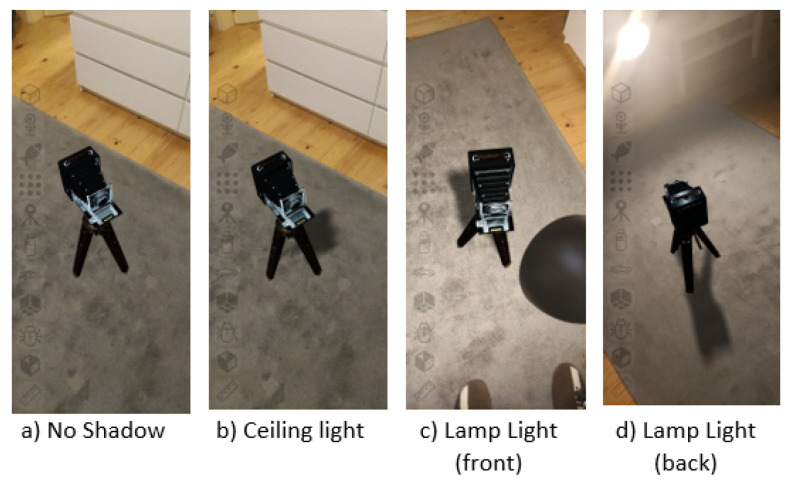
Shadow casting from virtual objects onto the floor plane.

Figure 10 presents the shadow-casting results, where it is possible to observe that the shadow was cast based on the direction of the main light source. In the example presented, the main light source could either be the ceiling light (present in a and b) or the desk lamp (visible in c and d).

To ensure good performance, shadow maps are typically rendered with a resolution considerably lower than the expected shadow pixel density in the screen space and to improve their perceived quality, a filtering method can be used. We tested 3 different algorithms for shadow map filtering: percentage-closer shadows (PCS) [38], variance shadow maps (VSM) [39] and percentage-closer soft shadows (PCSS) [40].

**Figure 11:**
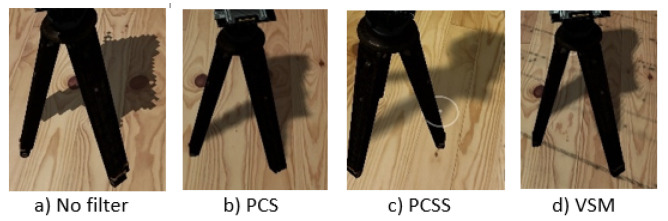
Shadow map filtering methods tested with resolution of 1024×1024 pixels.

To evaluate the performance and quality of the shadow-mapping mechanism based on its resolution and filtering algorithm, we tested multiple configurations and measured the performance for each one, and we selected a shadow area of 5×5 m, equals 25 square meters. The performance results are presented in Table 1. Examples for each filtering method are presented in Figure 11. Based on these results, we selected a resolution of 1024×1024 pixels for the shadow map of the directional light using PCSS filtering as the best compromise between quality and performance.

### 3.6. Physics Interaction

To provide an interaction between virtual and real world objects, depth information was used to create a simplified model of the real environment that was combined with the virtual scene object for the physics simulation.

For the physics simulation, the cannon.js rigid body physics engine was used. It was selected based on its performance through the usage of the SPOOK stepper [41] combined with a spatial indexation of physics elements. The physics simulation ran from a separate thread using a web worker. To share information with the main thread, data were packed into a shared buffer to prevent duplicated data entries between different workers.

**Figure 12:**
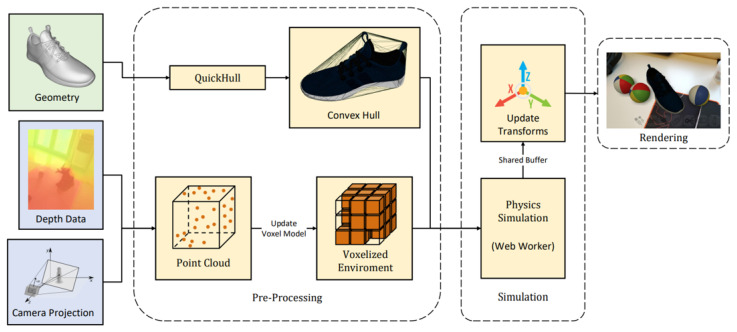
Physics pipeline for interaction with the environment: 3D geometries are used to generate convex hulls that result in better performance for the physics simulation. The environment is mapped using a probabilistic voxel grid model that is updated every frame; the simulation is performed on a separate web worker that communicates with the main thread using shared buffers to prevent duplicated data in memory.

The process for the physics simulation is represented in Figure 12; the process starts with a reconstruction of the real-world environment by projecting the depth data into a probabilistic voxel occupancy grid using a technique based on the work presented in [42].

The voxel occupancy grid consisted of a dense voxel volume that could be expanded dynamically as required. Voxel size should be adapted to the precision required. For the implementation, we selected empirically a voxel size of 5 cm. Voxels stored a weight value, which was compared against a threshold value to decide if the voxel was occupied by a real-work object or not.

Equation (1) represents how the system updates voxels contained in the camera volume, where *V_w_* is the voxel weight, *U_s_* is the update speed, from 0 to 1, and *H_t_* is the depth hit result with values 0 or 1.

(1) $$V_{p}^{\prime }=\left(V_{w}*\left(1-U_{s}\right)\right)+U_{s}*H_{t}$$

The depth was projected into a 3D space; each point had to hit a voxel in the grid; if not, the voxel grid needed to be expanded. For each point, all voxels close to the camera had a reduction in occupancy weight and the voxel hit by the point had its weight increased. Voxels outside of the view were not updated. To handle virtual objects and maintain a good computational performance of the physics simulation, simplified convex hulls were generated for each object geometry using the quick-hull algorithm [43].

Information from the probabilistic voxel model and convex hulls were provided to the physics engine, which updated the simulation state and the position of virtual objects before rendering each frame.

**Figure 13:**
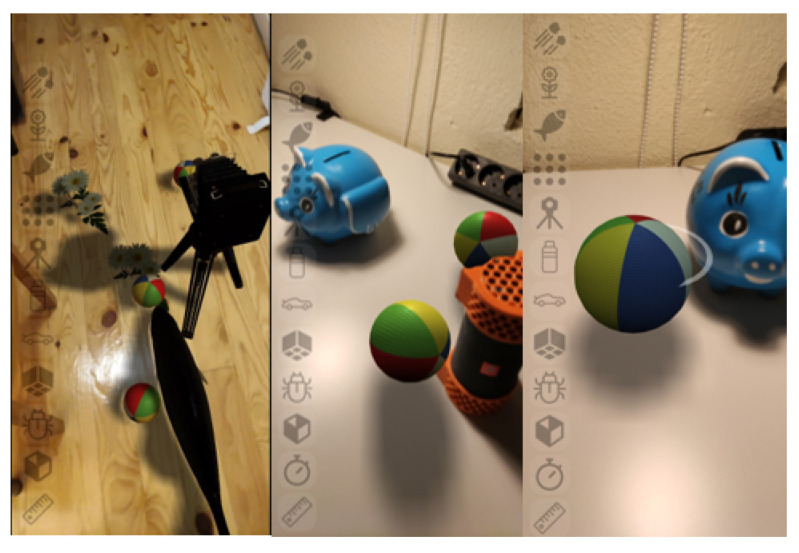
Example of physics objects (balls) collision with real objects modelled by a voxel volume model.

Figure 13 exemplifies the collisions between real and virtual objects. A gap between real and virtual objects due to the low resolution of the voxel grid was observed. Depending on the scenario, it might be necessary to adjust the model to prioritize noise removal or update the speed to accommodate dynamic objects. This would be mostly dependent on data quality. In the described case, given the considerable amount of noise, the noise removal was prioritized using a lower Us value.

### 3.7. Usage

The solution presented is distributed openly as a library and focuses on providing an abstraction layer for web AR with fallbacks available for non-AR devices. The code is available on GitHub at www.github.com/tentone/enva-xr (accessed on 12 February 2023).

**Figure 14:**
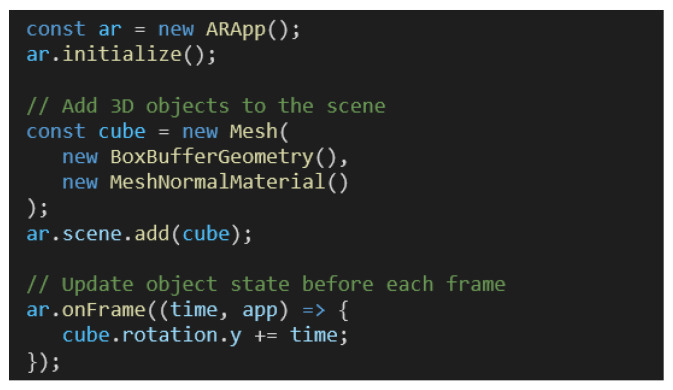
Code required for basic AR app with spinning cube.

The solution takes care of all initialization code required, so that developers can focus on the application development. Figure 14 presents the code required to add an object in a 3D scene with all features described in this document enabled by default.

The “ARApp” object provides access to all the features presented in the document. It checks for the existence of all WebXR features required in the device and manages the rendering of 3D objects and the physics simulation.

The features of the system can be toggled trough flags and developers can provide 3D objects to the “scene” attribute of the application instance. A state update of 3D objects can be performed in the “onFrame” method called before rendering each frame.

## 4. Performance Evaluation

To evaluate the performance of the framework, a simple implementation using only the environment-tracking functionality without any lighting or geometry effect was considered as a baseline, similarly to the solution proposed by [25].

**Figure 15:**
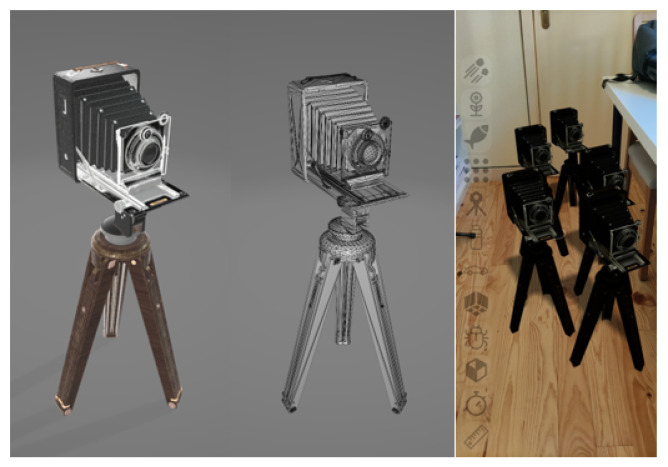
Antique camera model from Khronos glTF 2.0 sample repository.

A 3D model of an antique camera available under MIT’s permissive license was used; this model is shown in Figure 15 and is composed of 20,162 triangles, split across eight entities, and a total of six textures with a resolution of 2048×2048 pixels each, to encode surface properties: colour, normals, roughness, and metalness.

No optimizations were used during the tests and each group of entities used its own textures and geometries that were replicated in memory. All objects were placed under direct visibility, otherwise the framework would remove them from rendering. The device was placed on a fixed stand during the tests to ensure that the tracking process was under similar conditions.

**Figure 16:**
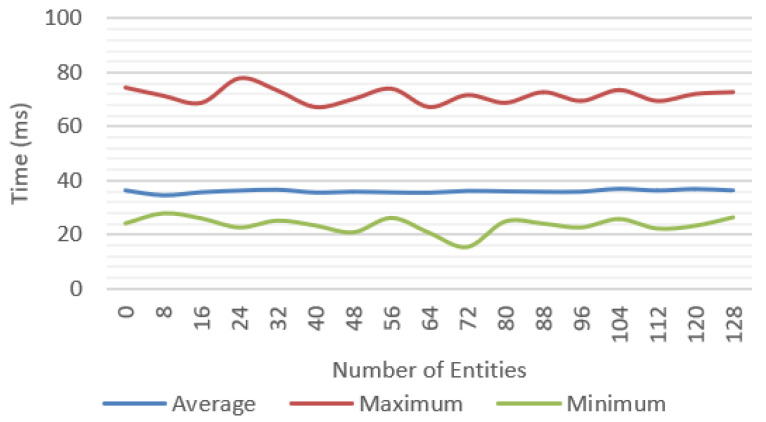
Frame time for base solution with environment tracking.

**Figure 17:**
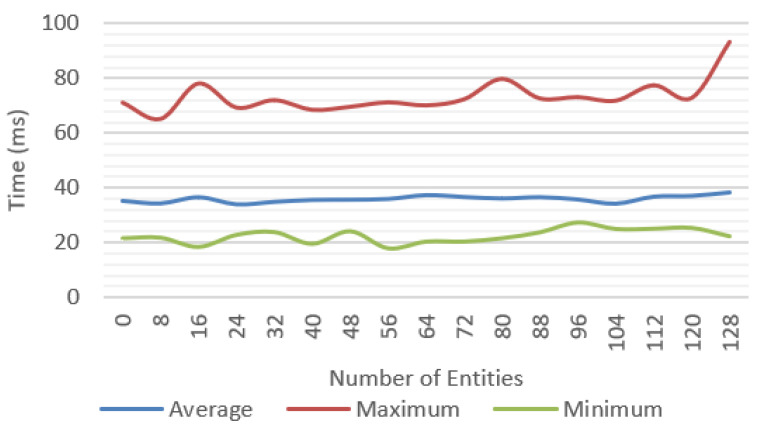
Frame time for tracking and geometry occlusion.

**Figure 18:**
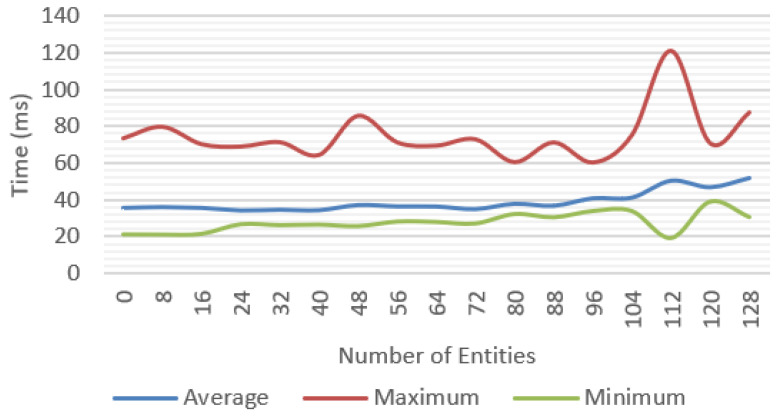
Frame time for tracking, geometry occlusion, and environment lighting.

**Figure 19:**
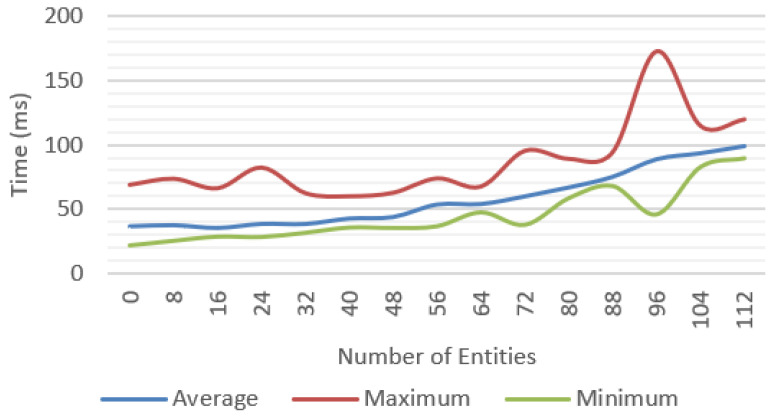
Frame time for the complete solution with tracking, geometry occlusion, lighting, and shadow casting.

Incremental tests were performed for each of the features proposed with different number of objects; for each scenario, the average, minimum, and maximum values were measured. We tested a baseline approach only with environment tracking (Figure 16), a geometry occlusion feature (Figure 17), PBR rendering (Figure 18), and finally, the system was tested with all the features (Figure 19).

**Figure 20:**
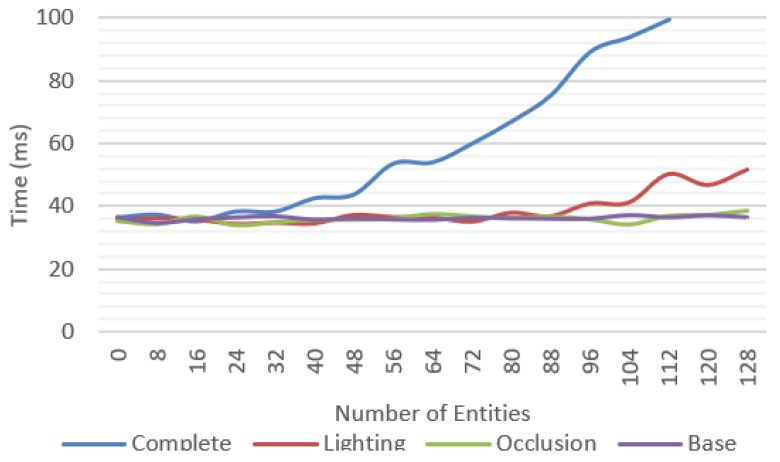
Average frame time for multiple test scenarios considering the total time between frames.

Figure 20 compares the average frame time for each feature and shows that the complete system was able to work at reasonable frame rates, at an average of 30 fps up to 40 entities and getting as low as 10 fps in the worst-case scenario.

A significant variation between minimum and maximum frame times could be observed. In the complete system test (Figure 19), we observed more than 100 ms in difference between these values.

For the most complex scenario, the device ran out of memory for more than 112 entities, caused by the quantity of texture data required for the textures and geometries. In a real scenario, this situation could be easily avoided by compressing and sharing the resources across the multiple entities that can be implemented in the solution.

**Figure 21:**
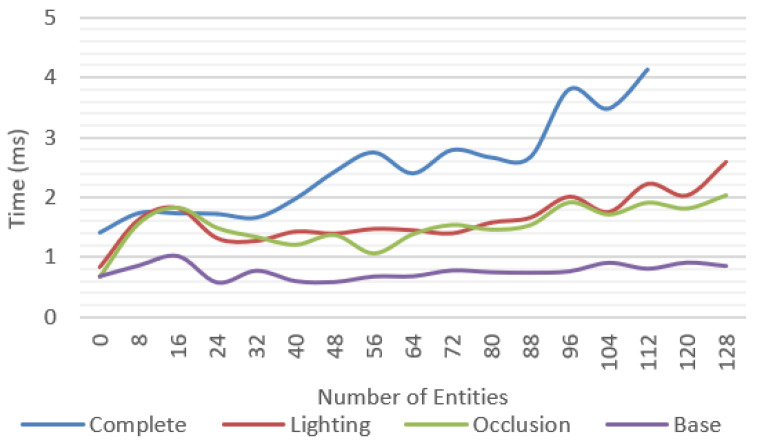
Average rendering time for the multiple test scenarios, not including the WebXR overhead.

All tests were repeated by measuring only the rendering time of the solution proposed without accounting for the WebXR overhead, considering only the time required to update the data structures and render an image to the screen. The results are presented in Figure 21 showing a maximum difference of up to 4.2 ms of additional processing time introduced by the proposed solution.

In a device using a dedicated depth solution this value is expected to be the major penalty introduced by the platform, easily allowing high-end devices to sustain higher frame rates.

**Figure 22:**
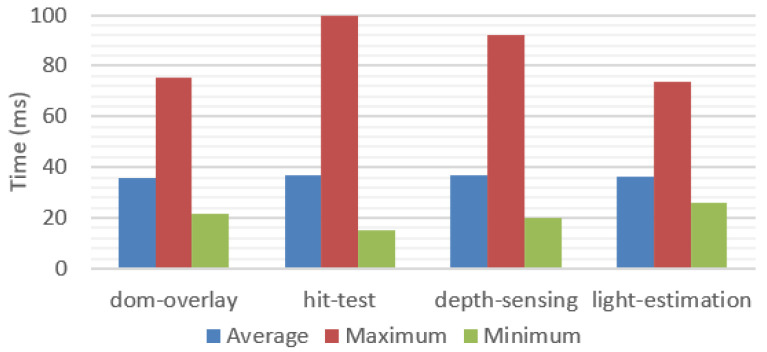
Performance values for each individual WebXR feature, without accounting for our framework processing overhead.

To understand better how the WebXR API impacted performance, a new test, independent from the framework, was prepared, evaluating the times for each one of the WebXR API modules necessary to access information used by the framework. Each feature was tested separately, and the results are presented in Figure 22.

The results suggested that no specific feature of the API imposed a higher performance limitation as the performance was similar independently of the features in use. A big gap between minimum and maximum values was observed, confirming again the substantial variation in processing time of the API.

## 5. Comparison

For comparison of the approach presented, we selected two solutions: DepthLab and MyWebAR. DepthLab uses the AR Core API and can be considered a representative of the state-of-art in mobile augmented reality [23]. MyWebAR is a commercially available solution for portable AR experiences, with minimal setup required and compatibility with the WebXR API.

### 5.1. DepthLab

**Figure 23:**
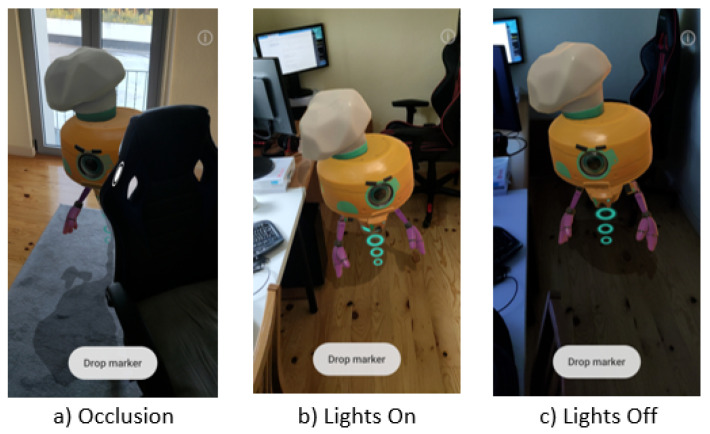
Rendering geometry occlusion and environment lighting using DepthLab.

DepthLab version 1.0.5 and the device presented in Section 3 were used for the results presented. Analysing the rendering capabilities of both solutions suggested that they offered similar results regarding occlusion and lighting capabilities as displayed in Figure 23. It was also possible to observe the same depth limitations, which was expected, as the same underlying techniques were used.

Although surface lighting used a similar technique with PBR materials, the proposed solution lacked environment maps, relying instead on a simplified spherical harmonics (SH) model, which, although not as detailed, provided a better consistency, as observed when comparing the results from Figure 23c, where the virtual object presented an orange tint and looked incoherent compared to the blue light of the environment and in Figure 9c, where the environment lighting colour was matched correctly.

Shadows were also obtained using different techniques; the proposed approach used shadow maps projected over planar surfaces with soft filtering, whereas the solution by [23] used hard shadows projected over a closed mesh (that was also used for the physics interaction).

**Figure 24:**
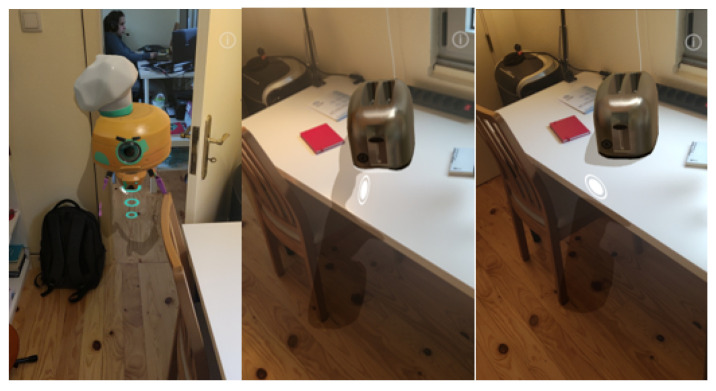
Shadow projection limitations using DepthLab: shadows are only partially displayed and cross surfaces.

The results obtained with the DepthLab solution also presented some issues as seen in Figure 24: the shadows were not always correctly projected and often occluded real-world objects. The shadow direction and style also did not match the surrounding objects, and there was sometimes a complete lack of shadows.

For the physics interaction, the solution presented combined a voxel occupancy grid approach, which was built and updated as a simplified but dynamic model of the environment with a simplified hull for the virtual objects and which allowed the physics simulation to run even for elements outside of the viewport.

**Figure 25:**
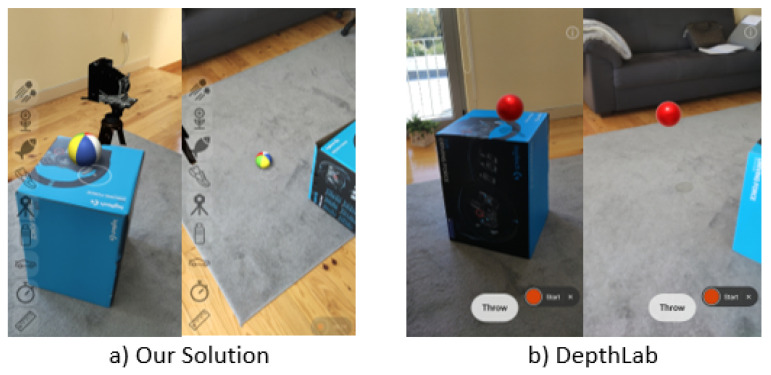
Physics simulation with floating objects after removing a cardboard box in DepthLab and objects falling in our solution.

The approach presented in DepthLab used a closed triangulated mesh updated in real time from the depth data available in the viewport, which did not cope well with physics elements leaving the screen space or interacting with the surface. Figure 25b shows that the simulation stopped once we placed a virtual object over a cardboard box. Once the objects hit the box, they became static and no longer reflected changes in the environment.

In contrast, the proposed solution (Figure 25a) updated the simulation for all objects, updating our voxel-based model according to environment changes. After removing the cardboard box, the object fell to the ground. A current limitation was the delay introduced since our system required some time to update the model (several frames were necessary to update the occupancy value).

### 5.2. MyWebAR

MyWebAR is a web-based commercial solution for AR experiences. It supports different types of AR projects based on image tracking, QR codes, and SLAM. For comparison, we created a SLAM project. In SLAM projects, MyWebAR can use WebXR to track the environment similarly to the solution presented.

**Figure 26:**
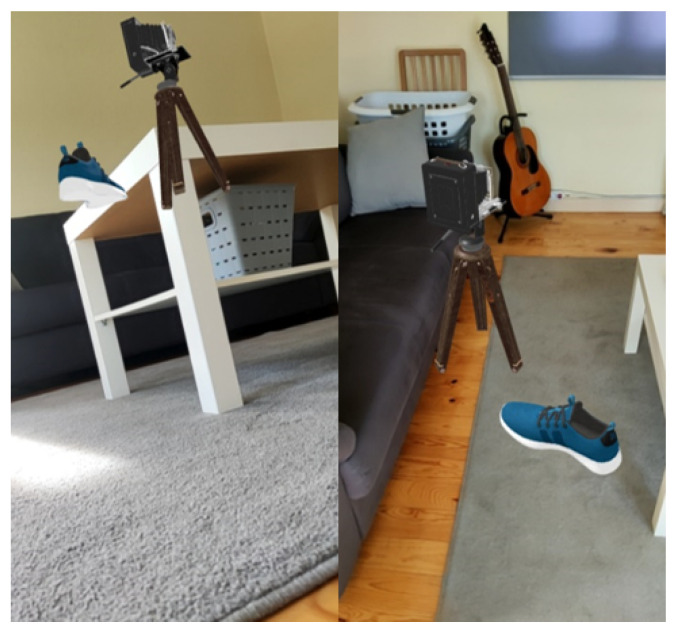
MyWebAR platform-rendering capabilities.

In Figure 26, we observe that MyWebAR lacked many of the rendering features introduced by our solution. For instance, occlusion was not handled correctly with objects rendered on top, and no shading was applied to the objects. We also observed a drift in the position of objects placed in the environment which might indicate that no anchoring mechanism was implemented in that solution.

## 6. Conclusions and Future Work

This paper presented a framework for environment-aware AR application based on the WebXR API providing a consistent and performant package paving the way for the creation of complex AR applications that can better reflect changes in the environment.

The novelty and a most significant advantage of the proposed approach resulted from the integration of multiple state-of-the-art AR features into a single performant package (as exemplified in Video S1) that could run on devices with vastly different hardware configurations.

At the time of this work, the specification for the WebXR API and the modules used (“depth”, “lighting-estimation”, “hit-test”, “anchors”, and “geo-alignment”) were in a proposal state, and their implementation was subject to changes in future releases of the Chrome web browser.

The framework presented is intended to be used by other developers and researchers. It allows users to quickly create a web AR application capable of environment interaction with minimal code required for setup.

While the WebXR API specification is still under development, it already offers a significant toolset for environment-aware mixed reality. The performance instability of the WebXR API probably caused by the unpredictability of the SLAM process used to build the environment model, poses some challenges for consistent performance in more complex AR applications, which was also observed in [21].

To further enhance the results of the work presented, real-time reflections could be introduced using environment cube-map information. It was not possible to integrate this feature because the API provided it as a preallocated WebGL texture that we were not able to integrate into the three.js rendering pipeline. An alternative algorithm for occlusion could be used to improve the visual fidelity of the solution; for instance, in Figure 1, the occlusion was not perfectly represented and one of the antique camera’s feet should have been hidden behind the white table leg.

Throughout the development of the proposed framework, it was noticed that memory limitations arose quickly, which was expected considering the complexity of the SLAM process used internally by ARCore to keep track of the device position and environment anchors.

To reduce the memory used for textures, which represent the largest chunk of memory, GPU-compressed formats should be used whenever possible and are supported by the proposed framework. To ensure compatibility with vendor-specific formats, the approach proposed by [44] provides a middleware format that can be transcoded to vendor-specific technologies and has a reduction of up to 96% in GPU memory usage when compared to uncompressed data.

## Figures and Tables

**Table 1:** Shadow map rendering performance with different resolutions and filtering methods.

Resolution	$256^{2}$ pxs	$1024^{2}$ pxs	$4096^{2}$ pxs	$8192^{2}$ pxs
Basic	1.10 ms	1.32 ms	1.37 ms	1.51 ms
PCF	1.12 ms	1.42 ms	1.97 ms	2.75 ms
PCF Soft	1.18 ms	1.53 ms	2.08 ms	3.42 ms
VSM	1.15 ms	1.67 ms	3.18 ms	5.42 ms

## Data Availability

Not applicable.

## Supplementary Materials

The following supporting information can be downloaded at: https://www.mdpi.com/article/10.3390/jimaging9030063/s1. Video S1: Functional demonstration of the features proposed by the framework.
